# SARS-CoV-2 Susceptibility of Domestic Animals and Wildlife in the Media Narrative

**DOI:** 10.3390/pathogens11111356

**Published:** 2022-11-15

**Authors:** Giovanni Di Guardo

**Affiliations:** General Pathology and Veterinary Pathophysiology, Veterinary Medical Faculty, University of Teramo, Località Piano d’Accio, 64100 Teramo, Italy; giodiguardo@libero.it; Tel.: +39-347-6317862

Since its discovery in December 2019 in China, severe acute respiratory syndrome coronavirus 2 (SARS-CoV-2) has caused over 630 million cases of human infection globally, along with almost 7 million deaths due to coronavirus disease 2019 (COVID-19) (World Health Organisation, WHO).

Despite the likely origin of SARS-CoV-2 from a primary animal source (*Rinolophus* spp. bats) [1], coupled with the different SARS-CoV-2 susceptibility levels shown in both domestic species and wildlife including ungulates and carnivores [2], the predominant narrative about SARS-CoV-2 infection and COVID-19 still neglects some crucial aspects. Indeed, inappropriate and distorted storytelling is still alive in the “COVID-19 media arena”, with special emphasis on the emergence of new viral “variants of concern” (VOCs) and “variants of interest” (VOIs). Within this context, it seems quite bizarre that, when dealing either with “past” (“alfa”, “beta”, “gamma” and “delta”) or “current” VOCs such as “omicron” (alongside its highly contagious and recently characterized “Centaurus”, “Chiron”, “Gryphon” and “Cerberus” subvariants), attention is given almost exclusively to “viral-human host interaction”. Noteworthily, at least 29 different animal species, both domestic and wild, have been hitherto deemed naturally and/or experimentally susceptible to SARS-CoV-2 infection. These include, among others, wild cetaceans such as bottlenose dolphins (*Tursiops truncatus*), based upon the homology level of their angiotensin-converting enzyme 2 (ACE2) viral receptor with the human one [3]. In this respect, while it should be firmly kept in mind, on one hand, that interhuman transmission is by far the prevailing mode of SARS-CoV-2 infection, it should be additionally emphasized, on the other hand, that clear-cut evidence of “man-to-animal viral spillover” and, to a lesser extent, of “animal-to-man viral spillback” events has been clearly documented. This is the case, for instance, of intensely bred mink from The Netherlands and Denmark, which, following SARS-CoV-2 infection transmitted from their breeders and keepers, returned a mutated virus (the so-called “cluster 5” VOC) to humans. Another interesting “animal-to-man viral spillback” episode is the one involving hamsters sold in Hong Kong pet shops, from which the “delta” VOC was transmitted to people, while a “highly divergent” SARS-CoV-2 strain has been recently transferred by an infected white-tailed deer (*Odocoileus virginianus*) to a man in Canada [4]. Still worthy of mention, the first documented case of “cat-to-human transmission” of SARS-CoV-2—caused once again by the “delta” VOC—has been recently described in a female veterinarian from Thailand [5]. Cases of infection by the “alfa” VOC were also reported in French cats and dogs suffering from heart disease, while the “delta” VOC was recovered from both symptomatic and paucisymptomatic dogs in Spain, with the “omicron” VOC being additionally identified in white-tailed deer from the States of New York and Ohio [4].

Among the wild animal species hitherto deemed susceptible to SARS-CoV-2 infection, a number of them appear to be increasingly threatened by extinction in terrestrial as well as marine ecosystems. This is an issue of great concern, which undoubtedly offers a strong argument for seriously taking into account the possibility of immunizing the aforementioned species against SARS-CoV-2. By doing so, in fact, we would not only protect animal biodiversity, thereby warranting adequate antiviral immunity to those SARS-CoV-2-susceptible wildlife species facing an increased extinction risk, but we would likely simultaneously contribute to reducing the virus’s circulation and, consequently, the appearance of new, highly transmissible and/or pathogenic VOCs. To this aim, the tremendous progress made in the production of the currently available anti-COVID-19 vaccines through the “revolutionary” messenger RNA (mRNA) technology should be viewed as a totally advantageous situation.

Notwithstanding all the above, the “SARS-CoV-2 media narrative” continues to be mostly, if not entirely, focused on the “virus-human host interaction”, thus forgetting that at least 70% of “emerging infectious diseases” have either a documented or suspected origin from one or more animal reservoirs, as clearly shown for SARS-CoV and MERS-CoV betacoronaviruses, and as largely postulated for SARS-CoV-2 also [1,6].

Sadly enough, this “storytelling asymmetry” has been also accompanied in Italy by an “operational asymmetry”: for two years, in fact, the “Italian Scientific and Technical Committee for the COVID-19 Pandemic”, popularly known by the acronym “CTS”, never included a single veterinarian among its 20 members (subsequently reduced to 12) [7]. This seems to be very far from the “One Health” concept and principle repeatedly promoted by the WHO as well as by the World Organisation for Animal Health, according to which, human, animal and environmental health are reciprocally and inextricably linked to each other.

*Errare Humanum est Perseverare autem Diabolicum!*
